# After Myocardial Ischemia-Reperfusion, miR-29a, and Let7 Could Affect Apoptosis through Regulating IGF-1

**DOI:** 10.1155/2015/245412

**Published:** 2015-12-30

**Authors:** Lei Wang, Xuehong Niu, Jihua Hu, Haijian Xing, Min Sun, Juanli Wang, Qiang Jian, Hua Yang

**Affiliations:** Department of Cardiology, Xi'an Children's Hospital, No. 69, Xiju Road, Lianhu, Xi'an, Shaanxi 710003, China

## Abstract

Cardiovascular and cerebrovascular ischemic disease is a large class of diseases that is harmful to human health. The primary treatment for the ischemic disease is to recover the blood perfusion and relieve the tissue hypoxia and the shortage of the nutrients in the supply of nutrients. In recent years, investigations found that IGF-1 has a protective effect on cardiovascular disease, especially in myocardial ischemia-reperfusion injury. Investigation into molecular mechanism of ischemia-reperfusion injury may offer potential targets for the development of novel diagnostic strategies. In this study we defined IGF-1 was differentially expressed in the I/R model of the Mus musculus and IGF-1 was the target gene of miR-29a and Let7f. After ischemia-reperfusion, the expression of miR-29a and Let7f increased, while the expression of IGF-1 decreased significantly in the animal model assay. Further studies have found that IGF-1 could inhibit cell apoptosis signaling pathway, thus protecting the reperfusion injury. These results provide new understanding of ischemia-reperfusion injury, with the hope of offering theoretical support for future therapeutic studies.

## 1. Introduction

Cardiovascular and cerebrovascular ischemic disease is a large class of diseases that is harmful to human health [1–3]. In most cases, ischemia-reperfusion can recover the function of organs, but sometimes ischemia-reperfusion will exacerbate structural damage and dysfunction [4]. This phenomenon is called ischemia-reperfusion injury (IR), and reperfusion injury may be more serious compared to ischemic injury alone. Cao et al. found that postconditioning obviously inhibits I/R induced lung injury by its antioxidant, anti-inflammatory, and antiapoptosis activities [5].

Insulin-like growth factor-1 (IGF-1) is an important growth factor, which plays an irreplaceable role in the regulation of cardiac structure and function [6]. In recent years, the researchers found that IGF-1 not only had insulin like function and mediated the growth hormone, but also was an apoptosis inhibitor of various types [7, 8]. As an inhibitor of apoptosis, IGF-1 functions by binding to the surface receptors of specific target cells. There has been lots of studies that confirmed that IGF-1 can reduce neuronal cell death in various damage [9], and IGF-1 has a protective effect in ischemic animal models [10]. Therefore, exploring the molecular mechanism of IGF-1 signaling pathways, especially in relation to cell apoptosis, may help to promote the treatment of cardiac disease.

MicroRNA (microRNA, miRNA) is a kind of endogenous single chain noncoding small RNA which has the tissue specificity or is expressed in specific developmental stage [11, 12]; length of 18~24 nucleotides, completely or incompletely complementary to the target mRNA, causes the target mRNA to degrade or inhibits its translation [13]. It plays an important role in physiological processes such as cell differentiation, cell cycle regulation, metabolism, and apoptosis. Approximately 3% of the human DNA sequences encode this class of highly conserved microRNA sequences. In the past, this kind of small RNA fragments is thought to be meaningless. With the development of technology, more and more research has found that this kind of small RNA fragment is important in the development of many kinds of life activities and important pathophysiologic events [14, 15]. And it has been reported that IGF-1 is a target gene regulated by Let7f.

In this study, we predict microRNA regulated IGF-1 by using software analysis. We construct the animal IR model and then explore the effect of microRNA on IGF-1 and apoptosis.

## 2. Materials and Methods

### 2.1. Identification of Differentially Expressed Genes and miRNAs

The mRNA and miRNA profiles data were collected from GEO database (http://www.ncbi.nlm.nih.gov/gds, GSE50884, GSE50886). The comparison of mRNA and miRNA profiles between heart tissues with and without ischemia-reperfusion (I/R) injury was performed with Limma package on R platform using raw microarray data, respectively. The cutline of significant differentially expressed mRNA and miRNAis is identified with fold change and *t*-test methods by the cutline |logFC| > 1 (fold change) and *P* value < 0.05 (*t*-test).

### 2.2. miRNA Target Genes Prediction

Mouse miRNA sequences were downloaded from the Rfam website (http://www.sanger.ac.uk/Software/Rfam). And 3′ UTR sequences for all mouse genes were retrieved using EnsMart. After that, repetitive elements in these sequences were masked using RepeatMasker. The target genes of miRNAs were predicted using TargetScan methods. The predicted target genes supported by all the three methods were selected for further analysis.

### 2.3. Animals

C57BL/6 mice, weighing 25–30 g, were provided with free access to food and water. Mice were randomly divided into 2 equal groups (10 mice each group): (a) sham (IR−); (b) IR+. Animal experiments were performed in accordance with the Guide for the Care and Use of Laboratory Animals. Every effort was made to reduce the suffering of animals.

### 2.4. Cell Culture and Transfections

H9C2 cells were cultured in DMEM supplemented with 10% FBS. Cell were transfected using Lipofectamine 2000 reagent (Invitrogen, Carlsbad, CA) according to the manufacturer's instructions. Mimic-miR-29a, mimic-Let7f, anti-miR-29a, and anti-Let7f were bought from Life Technologies, Beijing, China.

### 2.5. Ischemia-Reperfusion Model

Mice were anesthetized using 3% pentobarbital sodium and then supine fixation in the surgical table. Removed neck hair was cut 1 cm longitudinally, and the trachea was exposed. Insert tracheal intubation and connected with breathing machine. Removed chest hair was cut longitudinally 2 cm at the left side 0.5 cm of the midline of the chest, the chest was cut open, third to fifth ribs carefully were cut, the heart was exposed, and IR device was placed.

### 2.6. Quantitative Real-Time Reverse Transcription Polymerase Chain Reaction (RT PCR)

Quantitative real-time polymerase chain reaction (PCR) was performed as previously described by using Takara SYBR Green.

### 2.7. TUNEL (Terminal Deoxynucleotidyl Transferase dUTP Nick End Labeling) Assay

Broken DNA in the nucleus was labeled with TUNEL assay and visualized by fluorescence microscopy. TUNEL assay kit was bought from Roche.

### 2.8. Western Blotting Analysis

The cells were schizolysised in RIPA buffer containing protease inhibitors and phosphatase inhibitors. Protein concentration was determined colorimetrically by BCA assay. Protein lysates were separated by 12% SDS-PAGE electrophoresis and were transferred onto polyvinylidene difluoride (PVDF) membranes. After blocking with 5% BSA for 1 h, the membranes were incubated with antibodies at 4°C overnight, followed by incubation with secondary antibodies for 1 h. Then, the blots were visualized using ChemiDocTM XRS + System with Image LabTM Software (Bio-Rad Laboratories, Inc., USA). Experiments were performed 3 times.

### 2.9. Elisa Assay

Elisa kit was bought from Shanghai Enzyme-linked Biotechnology Co., Ltd. Elisa assay was carried out according to the manufacturer's instructions.

## 3. Results

### 3.1. IGF-1 Was Differentially Expressed in the I/R Model of the Mus Musculus and IGF-1 Was the Target Gene of miR-29a and Let7f

**Figure 1:**
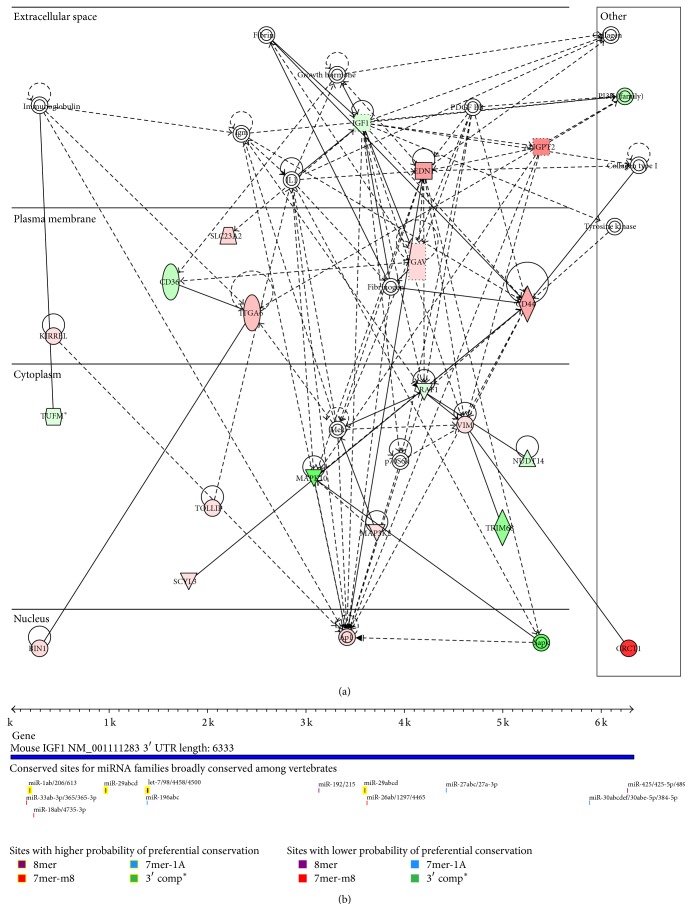
(a) IGF-1 related molecular network in I/R injury mouse. Upregulated genes are marked with red, while downregulated genes are marked with green. (b) TargetScan results of IGF-1.

We explored the expression value of IGF-1 in mouse heart with and without ischemia-reperfusion (I/R) injury and found that IGF-1 was significantly downregulated in mouse heart with I/R injury (Figure 1(a)). Then we performed miRNA-target prediction and differentially expressed miRNA identification. 135 miRNAs were found to be significantly upregulated in I/R injury. Among them, miR-29a and Let7f were predicted to bind the 3′ UTR of IGF-1 by TargetScan methods (Figure 1(b)). The results lead to the hypothesis that the low expression of IGF-1 maybe the results of upregulated miR-29a and Let7f.

### 3.2. IGF-1 Expression Decreased and miR-29a and Let7f Expression Increased after Ischemia-Reperfusion

**Figure 2:**
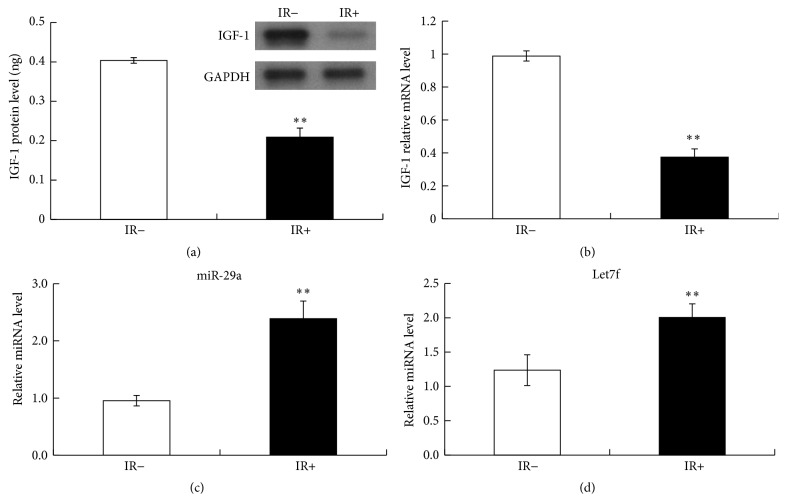
IGF-1 expression decreased after ischemia-reperfusion. (a) IGF-1 protein expression in IR− and IR+ group (*n* = 10) measured by Elisa and Western blot. (b) Quantitative analysis of relative IGF-1 protein expression in IR− and IR+ group measured by RT-PCR. MiR-29a (c) and Let7f (d) expression increased after ischemia-reperfusion.

There has been researches that showed that IGF-1 played a vital role in the inhibition of apoptosis, and IGF-1 could significantly reduce the apoptosis number of myocardial cells after ischemia-reperfusion injury. In this study, IGF-1 protein was quantified by ELISA, and IGF-1 mRNA was quantified by RT-PCR. As shown in Figure 2, IGF-1 expression remarkably decreased after ischemia-reperfusion in both protein level (Figure 2(a)) and mRNA level (Figure 2(b)). At the same time, the expression of miR-29a (Figure 2(c)) and Let7f (Figure 2(d)) was significantly upregulated.

### 3.3. miR-29a and Let7f Expression Increased after Ischemia-Reperfusion

We first identified by the literature search and in silico analysis two microRNAs, miR-29a and Let7f, as candidate regulators of IGF-1. Both published literature and bioinformatics analysis told us that suppressing miR-29a and Let7f could promote IGF-1 function in a variety of biological systems.

**Figure 3:**
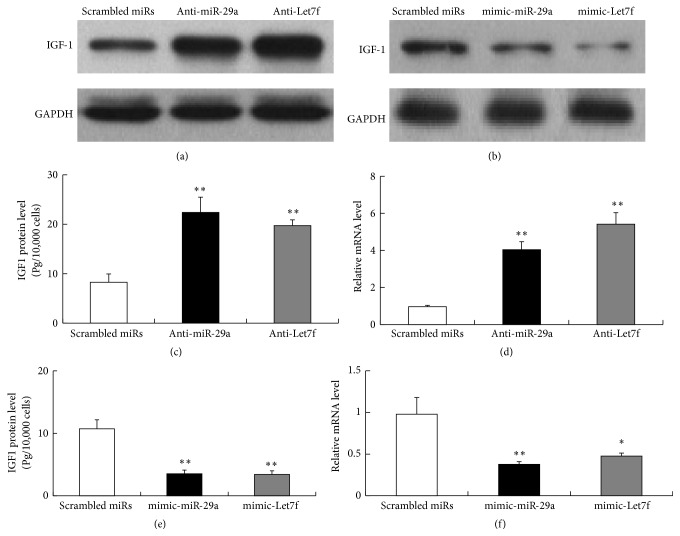
After transfection with anti-miR-29a and anti-Let7f, IGF-1 protein (a and c) and mRNA (d) level increased significantly. However, we found IGF-1 protein (b and e) and mRNA (f) level decreased after transfection with mimic-miR-29a and mimic-Let7f.

To further study the effect of miR-29a and Let7f on the function of IGF-1, we transfected H9C2 cells with scrambled miRs, mimic-miR-29a, mimic-Let7f, anti-miR-29a, and anti-Let7f, respectively. The ELISA and RT-PCR results showed that after transfection, IGF-1 protein and mRNA level increased several times compared with the scrambled miRs transfection group (Figures 3(a), 3(c), and 3(d)). In contrast, mimic-miR-29a and mimic-Let7f transfection significantly decreased the expression of IGF-1 (Figures 3(b), 3(e), and 3(f)).

### 3.4. miR-29a and Let7f Influenced IGF-1 Downstream Related Apoptosis Pathway

Currently, PI3K/Akt signaling pathway is considered as the classical pathway of IGF-1 inhibition of apoptosis [16]. Phosphatidylinositol-3 kinase, PI3K, is a heterodimer which is composed of p85 regulator subunit and P110 catalytic subunit and is the key link of signal transduction pathway in early signaling cascade. IGF-1 inhibits apoptosis and maintains the cell survival by activating the signaling pathway mediated by PI3K and its downstream Akt.

Caspases is the abbreviation for cysteine aspartate-specific protease. Among the 14 known caspases, the relationship of caspase-3, caspase-8, and caspase-9 with apoptosis was most closely. By PI3K-Akt pathway, IGF-1 activated Akt, and then Ser196 of Akt was phosphorylated, directly prevented caspase-3 activation, and inhibited apoptosis [17].

**Figure 4:**
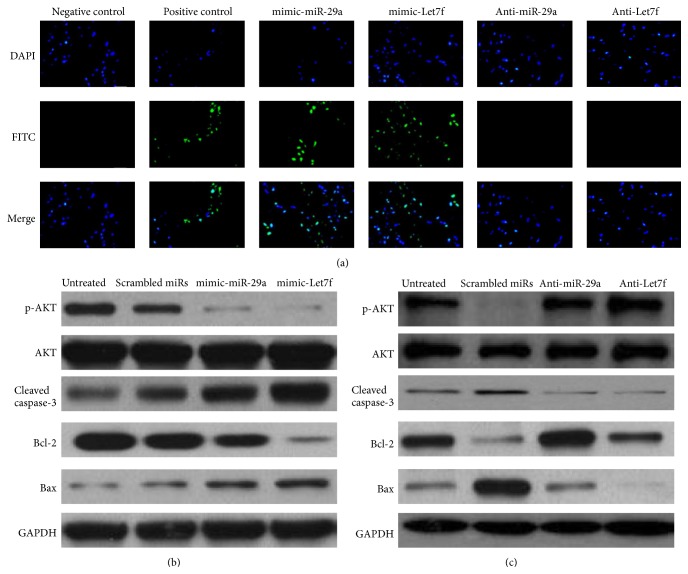
MiR-29a and Let7f influenced IGF-1 downstream related apoptosis pathway. (a) H9C2 cell apoptosis was detected by TUNEL assay. (b) We transfected cell with mimic-miR-29a and mimic-Let7f, then found the protein level of p-Akt and Bcl-2 decreased while caspase-3 and Bax increased. (c) After transfection with anti-miR-29a and anti-Let7f, Western blot results showed that p-Akt and Bcl-2 significantly increased, while caspase-3 and Bax protein significantly decreased.

In our study, after H9C2 cells were treated 48 hours with serum-free culture, we transfected H9C2 cells with scrambled miRs, mimic-miR-29a, mimic-Let7f, anti-miR-29a, and anti-Let7f, respectively. From TUNEL assay results (Figure 4(a)) we could see that mimic-miR-29a and mimic-Let7f transfection promoted cell apoptosis, while anti-miR-29a and anti-Let7f transfection inhibited apoptosis. Then we detected the change of phosphorylated Akt and cleaved caspase-3 protein expression. From the Western blot results, as shown in Figures 4(b) and 4(c), we found that the phosphorylated Akt significantly and antiapoptotic proteins Bcl-2 increased, and the amount of cleaved caspase-3 protein and proapoptotic protein Bax significantly decreased. These results indicated that after transfection with anti-miR-29a and anti-Let7f, the function of and miR-29a and Let7f was inhibited, and apoptosis was significantly decreased.

## 4. Discussion

Cardiovascular and cerebrovascular disease is a major disease that endangers human health and life. The primary treatment for the ischemic disease is to recover the blood perfusion and relieve the tissue hypoxia and the shortage of the nutrients in the supply of nutrients. Prevention and treatment of myocardial I/R injury have been studied for many years; control of reperfusion conditions, application of cell protective agent, and mobilizing the endogenous protection mechanism have achieved certain results [18–20]. However, the clinical application of myocardial ischemia protective measures still lacks breakthrough progress. Future direction is to clarify the core mechanism of I/R injury and then improve reperfusion therapy.

The existing studies have indicated that apoptosis is the essential component of the life activity of multicellular organisms, and it is the need of survival and exists throughout the total life cycle of the organism [21]. This process plays a key role in the development, formation, and homeostasis maintenance of tissues and organs. However, the imbalance of cell population quantity caused by excessive apoptosis is important reason for lots of clinical diseases [22, 23]. Therefore, it has important theoretical and practical significance to study the mechanism of apoptosis and regulation of apoptosis.

IGF-1 can regulate cell metabolism and promote its growth and development, in recent years, as a key regulator of cardiovascular disease [24]; IGF-1 is gradually valued. Increasing evidences suggest that IGF-1 has a protective effect on cardiovascular disease. IGF-1 is involved in the protection of I/R through the mechanism of cellular signal transduction and metabolic mechanism. The inhibition of apoptosis by IGF-1 has become a hot research hotspot [25], which is closely related to the occurrence of clinical diseases.

In this study, applied bioinformatics analysis method, we found that IGF-1 was differentially expressed in I/R model of the Mus musculus. Using Targetscan, mirSVR, and PicTar methods, there exist the binding sites of miR-29a and Let7f in the sequence of IGF-1. Therefore we predicted that IGF-1 is target gene regulated by the miR-29a and Let7f.

To confirm the ideas above we then implemented the animal model assay and found that, after I/R, the expression of miR-29a and Let7f increased, while the expression of IGF-1 decreased significantly.

As an inhibitor of apoptosis, IGF-1 can interact with multiple signaling pathways to play a role in inhibiting apoptosis. We used serum starvation method to induce apoptosis, and then we transfected the cell with anti-miR-29a and anti-Let7f, respectively. Western blot results showed the level of p-Akt decreased and cleaved caspase-3 was upregulated, whereas the anti-miR-29a and anti-Let7f transfection group was just the opposite and thus inhibited the apoptosis and protected from the I/R injury.

Although regulation mechanism of IGF-1 on apoptosis inhibition has not been fully elucidated and there may still exist many unknown signaling pathways and regulatory microRNA played a role, IGF-1 may be seen as a target which provides a new way for the treatment of related diseases.
